# Patterns of health literacy and influencing factors differ by age: a cross-sectional study

**DOI:** 10.1186/s12889-025-22838-6

**Published:** 2025-04-26

**Authors:** Da Hae Kwon, Young Dae Kwon

**Affiliations:** 1 Department of Public Health, Graduate School, The Catholic University of Korea, Seoul, 06591 Korea; 2 Incheon Public Health Policy Institute, Incheon, 22532 Korea; 3 Department of Humanities and Social Medicine, College of Medicine, The Catholic University of Korea, 222 Banpo-Daero, Seocho-gu, Seoul, 06591 Korea; 4 Catholic Institute for Public Health and Healthcare Management, The Catholic University of Korea, Seoul, 06591 Korea

**Keywords:** Health literacy, Age differences, Influencing factors, Health disparities, Korea

## Abstract

**Background:**

Individuals with better health literacy communicate more effectively with healthcare providers, adhere better to medical instructions, and manage their health more successfully. While interest in health literacy is increasing, there is still a lack of comprehensive analysis that includes all age groups. In this study, we examine differences in health literacy and in health literacy-related factors between the elderly and other age groups.

**Methods:**

This study used data from the 2020–2021 Korea Health Panel and the 2021 health literacy supplementary survey. The survey included 7,910 participants aged 19 and older and employed the HLS-EU-Q16 tool to measure health literacy. Sociodemographic, health behavior, and healthcare utilization variables were analyzed. Statistical analyses, including chi-square tests, t-tests, and multiple regression analyses, explored the relationships between health literacy and factors influencing it across different age groups.

**Results:**

Health literacy scores decreased with age, with the average score for all participants being 10.96. Specifically, the young group scored higher (14.10), the middle-aged group scored moderately (12.43), and the older group scored the lowest (8.34). We identified several factors influencing health literacy that were consistently significant across age groups, including education, income, self-rated health, and quality of life. Notably, in the elderly group, health-promoting behaviors like walking were associated with health literacy, and those with low education or chronic diseases faced the most significant challenges in health literacy.

**Conclusions:**

We found that health literacy declined significantly as adults grew older. In addition, factors related to health literacy differed by age group. These results highlight the need for tailored interventions to meet the specific needs of each age group.

**Supplementary Information:**

The online version contains supplementary material available at 10.1186/s12889-025-22838-6.

## Background

Health literacy (HL) is defined as the ability of individuals to obtain, process, and understand the basic information and services they need to make appropriate decisions about their health and healthcare [1]. This definition refers to functional HL [2], distinct from linguistic HL, which is defined as the ability to comprehend commonly used terms and phrases related to health, disease, or the body in a given language context [3]. HL is a major social determinant of health and has been reported to affect health outcomes, as well as health promotion, disease prevention, and healthcare utilization [4–7]. This concept is essential for individuals to communicate with professionals about their health conditions, to acquire necessary health and medical information, and to fully understand and follow medical instructions or education [8], and thus HL and the health of individuals and populations are closely related. In particular, functional HL has been identified as a more powerful predictor of health outcomes than socioeconomic status, age, and race [9].

Individuals with low HL often face difficulties in communicating with healthcare providers, which leads to poor adherence to medication and treatment, as well as challenges in managing chronic diseases and practicing health-promoting behaviors. Consequently, this can result in increased healthcare costs, higher hospitalization rates, and ultimately, higher mortality rates due to disease [10–14]. Therefore, HL skills are necessary to maintain and properly manage health [15]. For this reason, interest in this ability has increased, leading to more research exploring its influencing factors [16–20]. Overall, factors found to consistently influence HL include the age and education level of the individual [21]. Specifically, older individuals and those with lower levels of education tend to exhibit lower HL levels, likely due to reduced exposure to educational opportunities and limited access to health information resources [22].

Some studies have explored the determinants of HL in various age groups and populations [6, 10, 11, 23–25]. Beyond the findings that older adults have lower HL than young or middle-aged adults, studies have also suggested that HL-related factors may differ considerably by age group. In younger and middle-aged adults, HL is primarily associated with educational attainment, income, employment status, and digital information-seeking behavior [6, 10, 11]. In contrast, among older adults, HL is more closely linked to cognitive functioning, chronic disease burden, walking behavior, and access to a usual source of care [6, 23–25].

Many prior studies have focused on a specific age group - such as adolescents [11, 17] or older adults [19, 26] - rather than addressing HL across the general adult population. Much of the existing research has also targeted individuals with specific health conditions, such as cancer [11], heart failure [27, 28], or hypertension [29], thereby limiting the generalizability of the findings. Accordingly, a need remains to investigate HL across the general adult population using representative data. Since HL determinants may vary substantially across age groups, analyzing the adult population as a single group may obscure important age-specific patterns. It is essential to examine HL within the general population and to identify how HL-related factors differ across age groups to design age-appropriate strategies for health promotion and disease prevention. Particularly, in the context of rapidly aging populations worldwide, evaluating influences on HL and implementing policy efforts specific to the elderly is required to improve their health management capabilities and quality of life [26, 30].

This study aims to examine differences in HL patterns and associated factors across distinct age groups using nationally representative data and a structured measurement tool. By providing empirical evidence that reflects the diverse determinants of HL across the adult population, it is intended to contribute to the development of targeted, age-specific health literacy interventions.

## Methods

### Data

This study is descriptive research aimed at determining levels of HL and exploring factors related to HL across different age groups. The analysis utilized data from the 2020–2021 Korea Health Panel (KHP) annual survey and the health literacy supplementary survey conducted alongside the 2021 KHP. The KHP data comprehensively covers healthcare utilization behavior, expenses, and the factors influencing healthcare use, such as respondents’ health status and socioeconomic status. It was developed by a consortium that includes the Korea Institute for Health and Social Affairs and the National Health Insurance Service. The KHP data sample was selected using regional stratification variables from the 2005 Korean Population and Housing Census and has been tracked annually since 2008. Participants included individuals aged 19 and older, regardless of employment status, and were selected to represent the general population. The data consists of a cross-sectional time series, including individual differences (cross-sectional) and variations over time. The term “cross-sectional time series data” accurately characterizes the KHP data, as it combines observations from different individuals at a single time point with repeated measures for the same individuals over multiple years [31]. The survey is conducted annually by trained surveyors based on self-reported questionnaires and face-to-face interviews. It investigates healthcare utilization, including inpatient, outpatient, and emergency department visits, as well as information about household and household members. While the KHP is designed as a panel survey, this study utilized data from a single wave (2020–2021) and conducted a cross-sectional analysis.

The 2020–2021 annual data used in this study is part of the second data period of the KHP survey, which began in 2020. The second panel was designed to maintain national representativeness and utilized the 2016 registration census as the sampling frame. 6,190 households and 11,423 people responded to the 2020 survey, and 5,878 households and 10,931 people responded to the 2021 survey. This study utilized the 2020–2021 annual data to ensure alignment with the health literacy supplementary survey’s time frame, enhancing the analysis’s validity. However, the COVID-19 pandemic during this period may have influenced healthcare utilization and HL, as well as the data collection process, such as limitations on face-to-face interviews or changes in respondent behavior.

The KHP health literacy supplementary survey was conducted in 2021 to monitor the HL of the Korean population. It was conducted with the same respondents as the regular KHP survey to ensure consistency with previous KHP data. Previous HL studies in Korea have tended to have small sample sizes and unrepresentative samples. About three-quarters of studies published between 2010 and 2020 analyzed fewer than 500 participants, and more than two-thirds utilized non-representative samples [32]. The KHP health literacy survey is a comprehensive measure of this ability and has the advantage of exploring relationships using a wide range of variables, including demographic characteristics, health outcomes, healthcare utilization, and healthcare expenditures [23]. The study analyzed 9,530 individuals aged 19 and older who participated in the KHP health literacy supplemental survey between March and July 2021. The final analysis included a total of 7,910 participants, excluding 1,620 individuals who did not respond to the following items: healthcare utilization (*n* = 1,482), private health insurance status (*n* = 95), high-risk drinking (*n* = 38), current smoking (*n* = 4), and type of health coverage (*n* = 1).

### Variables and measurements

The dependent variable was HL, which was measured using the HLS-EU-Q16 tool [33]. Among various tools for measuring HL, the European Health Literacy Survey Questionnaire (HLS-EU-Q) is based on Sørensen’s comprehensive definition of this concept and is used to measure the comprehensive HL of individuals across diverse populations. HLS-EU-Q16 is composed of 16 items, including items about healthcare (seven items), disease prevention (five items), and health promotion (four items) from HLS-EU-Q47, which has 47 items [33]. The HLS-EU-Q16 is a shortened version of the HLS-EU-Q47. Therefore, it has a more limited conceptual representation, but it has been translated into several languages and confirmed to have valid psychometric properties [34]. The HLS-EU-Q16 has explicitly been validated for the Korean population, confirming its reliability and relevance in assessing HL within this demographic [23]. In the KHP survey, respondents answered each item of the HLS-EU-Q16 with “very difficult,” “difficult,” “easy,” “very easy,” or “do not know.” However, in this study, these responses were dichotomized into “easy” (1 point) and “difficult” (0 points), with “do not know” treated as a missing value. The final score of the HLS-EU-Q16 was calculated by summing the values of each item [34]. If there was a missing value for any response, the final score was treated as missing. The final score ranges from 0 (no/low HL) to 16 (high HL). These scores were categorized as inadequate (0–8 points), problematic (9–12 points), and adequate (13–16 points) [34].

Independent variables were selected from the KHP data based on previous research on HL, encompassing sociodemographic characteristics [4, 6, 10, 18, 19, 21], economic characteristics [10, 18, 19], health behaviors [4, 10, 11], healthcare utilization [6, 9, 14], and health status variables [5–7, 10, 18, 19, 26]. Multicollinearity among the independent variables was examined to finalize the selection. Sociodemographic and economic characteristics included gender, age, marital status, education level, income level, type of health coverage, private health insurance enrollment, and employment status. The status of supplementary private health insurance enrollment was assessed based on the year preceding the HL survey date, while all other variables were referenced as of December 31, 2020. Participants were classified into age groups: young adults (19–44 years), middle-aged adults (45–64 years), and older adults (65 years and older). Marital status was categorized as married (with a spouse), divorced/separated/widowed, and not married (single). Educational level was classified as elementary school or below, middle/high school, and college or above. Income level was measured as equivalized monthly household income, which is a household’s monthly income divided by the square root of the number of household members. The types of health coverage were categorized into National Health Insurance (NHI) and Medical Aid. Private health insurance enrollment status was classified as either enrolled or not enrolled. Employment status was dichotomized into participation (wage worker, self-support/public/senior/desired worker, employer, self-employed) and non-participation (unpaid family worker, economically inactive population).

Among health behavior-related characteristics, we selected current smoking, high-risk drinking, and walking. Current smokers were defined as individuals who were currently smoking, including cigarettes and nicotine-containing e-liquids, at the time of the health literacy survey. High-risk drinkers were defined as those who consumed seven or more glasses (or approximately five cans of beer) on a single occasion for men and five or more glasses (or approximately three cans of beer) for women at least twice per week in the past year prior to the survey. Walking practitioners were defined as those who walked for at least 30 min or more once daily, five days a week, based on the most recent week before the survey date.

Health status and healthcare utilization included self-rated health (SRH), number of chronic diseases, health-related quality of life (HRQOL), disability, number of outpatient visits and hospitalization experience, and usual source of care [18, 24, 35, 36]. SRH status was classified into five levels: very good, good, fair, poor, and very poor. The number of chronic diseases was categorized based on the 31 chronic diseases into three groups: no chronic disease, one chronic disease, and two or more chronic diseases. The EuroQoL 5 Dimension (EQ-5D) tool measured HRQOL. The EQ-5D consists of five questions about current health status: mobility, self-care, usual activities, pain/discomfort, and anxiety/depression, with three response options for each question. Participants’ responses were recorded based on the calculation method and standards provided by the Korea Disease Control and Prevention Agency and analyzed as continuous variables. Disability status was classified into disabled and non-disabled. Healthcare utilization was examined by the number of outpatient visits and hospitalization experiences from January 1 to December 31, 2021. The number of outpatient visits was treated as a continuous variable, while hospitalization experience was dichotomized into experienced and not experienced. The usual source of care was classified as ‘yes’ if there was a regular institution or doctor for treatment and ‘no’ if there was neither. Variables related to health status and healthcare utilization, excluding the number of outpatient visits and hospitalization experience, were based on the most recent year as of the date of the health literacy survey.

### Statistical analysis

Descriptive statistical analyses, including frequency, percentage, mean, and standard deviation, were conducted to identify the general characteristics of the study subjects. A chi-square test was used to examine distributions between age groups and other variables. T-tests and ANOVA were used to examine differences in HL according to socio-demographic and economic characteristics, health behaviors, health status, and healthcare utilization across different age groups. A multiple regression analysis was conducted to examine the influence of sociodemographic and economic characteristics, health behaviors, health status, and healthcare utilization on HL across different age groups. All statistical tests were two-sided and conducted at a significance level (α) of 0.05. Analyses were performed using SAS ver. 9.4 (SAS Institute Inc., Cary, NC, USA).

### Ethics

The Institutional Review Board of the Catholic University of Korea (MC24ZASI0109) approved this study with a waiver for informed consent because the data were obtained from a public data repository.

## Results

Of 7,910 participants, the majority (44.1%) were aged 65 and older, followed by 35.6% in the 45–64 age group and 20.3% in the 19–44 age group. Educational level varied significantly, with nearly half (49.7%) of participants aged 65 and older having an ‘elementary school or below’ level of education, compared to almost none (0.3%) in the youngest age group. Private health insurance coverage declined with age, with approximately 90% of participants in the 19–44 and 45–64 groups being insured, compared to only 53.1% in the 65 and older group. Economic activity followed a similar trend, with participation rates highest in the 19–44 age group (68.1%) and lowest in the 65 and older group (42.4%). Health behaviors and status indicators revealed additional age-related patterns. Participants aged 65 and older reported the highest walking rate (48.7%) but the lowest rates of current smoking (9.6%) and high-risk drinking (4.1%). Health status declined with age as the proportion of individuals reporting good health decreased, while those with two or more chronic conditions increased. The average HL score was 10.96 across all participants, showing an apparent decline with increasing age (Table 1).

**Table 1:** General characteristics of study samples by age group

			Age group						
	All (*n* = 7,910)		19–44 (*n* = 1,608)		45–64 (*n* = 2,815)		65+ (*n* = 3,487)		
Variable	n	%	n	%	n	%	n	%	χ^2^ by age group
Gender									2.33
Male	3,369	42.6	658	40.9	1,208	42.9	1,503	43.1	
Female	4,541	57.4	950	59.1	1,607	57.1	1,984	56.9	
Marital status									2,004.23^***^
Married	5,715	72.3	1,004	62.4	2,277	80.9	2,434	69.8	
Divorced/separated/widowed	1,481	18.7	45	2.8	415	14.7	1,021	29.3	
Not married	714	9.0	559	34.8	123	4.4	32	0.9	
Education Level									3,508.29^***^
Elementary school or below	2,001	25.3	4	0.3	263	9.3	1,734	49.7	
Middle/high school	3,442	43.5	356	22.1	1,618	57.5	1,468	42.1	
College or above	2,467	31.2	1,248	77.6	934	33.2	285	8.2	
Healthcare coverage									49.29^***^
National Health Insurance	7,561	95.6	1,581	98.3	2,701	96.0	3,279	94.0	
Medical Aid	349	4.4	27	1.7	114	4.1	208	6.0	
Having private health insurance									1,447.63^***^
Yes	5,867	74.2	1,487	92.5	2,528	89.8	1,852	53.1	
No	2,043	25.8	121	7.5	287	10.2	1,635	46.9	
Economic activity									576.82^***^
Yes	4,543	57.4	1,095	68.1	1,969	70.0	1,479	42.4	
No	3,367	42.6	513	31.9	846	30.1	2,008	57.6	
Current smoking									121.34^***^
Yes	1,146	14.5	294	18.3	518	18.4	334	9.6	
No	6,764	85.5	1,314	81.7	2,297	81.6	3,153	90.4	
High-risk drinking									194.50***
Yes	723	9.1	223	13.9	358	12.7	142	4.1	
No	7,187	90.9	1,385	86.1	2,457	87.3	3,345	95.9	
Walking									7.20^*^
Yes	3,740	47.3	718	44.7	1,325	47.1	1,697	48.7	
No	4,170	52.7	890	55.4	1,490	52.9	1,790	51.3	
Self-rated health									683.35^***^
Very good	246	3.1	88	5.5	76	2.7	82	2.4	
Good	2,511	31.7	723	45.0	990	35.2	798	22.9	
Fair	3,448	43.6	645	40.1	1,369	48.6	1,434	41.1	
Poor	1,590	20.1	147	9.1	361	12.8	1,082	31.0	
Very poor	115	1.5	5	0.3	19	0.7	91	2.6	
Number of chronic diseases									3,060.36^***^
0	2,739	34.6	1,280	79.6	1,146	40.7	313	9.0	
1	1,764	22.3	267	16.6	816	29.0	681	19.5	
≥ 2	3,407	43.1	61	3.8	853	30.3	2,493	71.5	
Disability									223.74^***^
Yes	594	7.5	27	1.7	137	4.9	430	12.3	
No	7,316	92.5	1,581	98.3	2,678	95.1	3,057	87.7	
Hospital admission									113.14^***^
Yes	1,142	14.4	146	9.1	332	11.8	664	19.0	
No	6,768	85.6	1,462	90.9	2,483	88.2	2,823	81.0	
Usual source of care									771.53^***^
Yes	5,015	63.4	639	39.7	1,634	58.1	2,742	78.6	
No	2,895	36.6	969	60.3	1,181	42.0	745	21.4	
	Mean	SD	Mean	SD	Mean	SD	Mean	SD	F
Monthly household income (1,000 KRW)	2,323.41	1,713.49	2,976.28	1,677.64	2,764.25	1,842.94	1,666.46	1,355.31	528.33^***^
Quality of life (EQ-5D)	0.94	0.10	0.98	0.05	0.96	0.07	0.91	0.12	423.88^***^
Number of outpatient visits	19.81	23.02	10.10	13.60	15.93	19.81	27.41	26.21	411.89^***^
Health literacy levels	10.96	4.79	14.10	2.94	12.43	4.06	8.34	4.65	1,337.46^***^

*p < 0.05. **p < 0.01. ***p < 0.001

KRW, Korean won; SD, standard deviation

In all age groups, variables showing significant differences according to HL levels were education level, type of healthcare coverage, participation in economic activities, and SRH. HL levels were higher among those with higher levels of education, those covered by NHI, those participating in economic activities, and those who reported their SRH as ‘good’ or ‘very good.’ Some variables influenced HL in specific age groups. In the 19–44 age group, those without a usual source of care had higher HL levels. In both the 45–64 and 65 and older age groups, men, individuals who had private health insurance, those with fewer chronic diseases, or those without a disability had higher HL levels. Specifically for the 45–64 age group, HL was highest among those with a spouse, while those who had been hospitalized showed lower HL. In the 65 and older group, HL was higher among those who had never been married, as well as those who were current smokers, engaged in high-risk drinking, or practiced walking for at least 30 min five days a week (Table 2).

**Table 2:** Bivariate analysis on health literacy by age group

				Age groups								
	All (*n* = 7,910)			19–44 (*n* = 1,608)			45–64 (*n* = 2,815)			65+ (*n* = 3,487)		
Variable	Mean	SD	t/F	Mean	SD	t/F	Mean	SD	t/F	Mean	SD	t/F
Gender			-8.90^***^			-1.30			-4.20^***^			-10.81_***_
Male	11.51	4.61		14.21	2.93		12.80	4.03		9.30	4.63	
Female	10.56	4.88		14.02	2.94		12.15	4.07		7.61	4.52	
Marital Status			326.54^***^			2.57			7.82^***^			53.08^***^
Married	11.24	4.62		14.01	2.98		12.58	3.94		8.85	4.62	
Divorced/separated/widowed	8.60	4.95		13.51	3.39		11.78	4.35		7.10	4.47	
Not married	13.65	3.62		14.29	2.81		11.93	5.02		9.00	4.41	
Education Level			1622.98^***^			41.37^***^			152.24^***^			314.06^***^
Elementary school or below	6.92	4.24		2.75	2.75		9.07	4.64		6.60	4.08	
Middle/high school	11.30	4.40		13.53	3.59		12.24	4.00		9.72	4.46	
College or above	13.78	3.25		14.29	2.62		13.70	3.35		11.82	4.46	
Healthcare coverage			-9.99^***^			-2.13^*^			-4.95^***^			-3.87^***^
National Health Insurance	11.08	4.75		14.14	2.85		12.53	3.96		8.41	4.67	
Medical Aid	8.48	5.02		11.74	5.82		9.95	5.52		7.15	4.17	
Having private health insurance			-30.95^***^			-1.76			-5.29^***^			-13.43^***^
Yes	11.95	4.32		14.14	2.84		12.59	3.91		9.31	4.51	
No	8.14	4.95		13.51	3.86		10.98	4.99		7.24	4.56	
Economic activity			-18.12^***^			-2.38^*^			-6.36^***^			-4.14^***^
Yes	11.80	4.47		14.22	2.70		12.77	3.82		8.72	4.65	
No	9.84	4.97		13.82	3.37		11.64	4.49		8.06	4.62	
Current smoking			-7.27^***^			1.75			-0.43			-3.60^***^
Yes	11.87	4.50		13.79	3.46		12.50	4.12		9.21	4.67	
No	10.81	4.82		14.17	2.80		12.41	4.05		8.25	4.63	
High-risk drinking			-10.85^***^			-0.44			-1.83			-2.88^**^
Yes	12.56	4.07		14.17	2.68		12.80	4.00		9.44	4.36	
No	10.80	4.83		14.08	2.98		12.38	4.07		8.29	4.65	
Walking			-4.99^***^			-0.96			-1.12			-8.45^***^
Yes	11.25	4.63		14.17	2.87		12.52	3.97		9.01	4.64	
No	10.71	4.92		14.03	2.99		12.35	4.14		7.70	4.56	
Self-rated health			247.89^***^			12.32^***^			28.03^***^			70.39^***^
Very good	12.00	5.36		14.74	2.65		12.62	4.81		8.49	6.08	
Good	12.37	4.23		14.38	2.50		12.99	3.85		9.78	4.64	
Fair	11.29	4.48		13.94	3.14		12.53	3.76		8.92	4.49	
Poor	8.22	4.88		13.20	3.56		10.70	4.84		6.72	4.23	
Very poor	6.13	4.14		8.00	4.95		8.00	5.51		5.64	3.65	
Number of chronic diseases			770.93^***^			2.64			37.85^***^			53.55^***^
0	13.33	3.62		14.18	2.83		13.13	3.65		10.58	4.74	
1	11.23	4.71		13.75	3.42		12.35	4.14		8.90	4.80	
≥ 2	8.93	4.75		13.84	2.72		11.56	4.34		7.91	4.49	
Disability			14.84^***^			1.98			5.17^***^			5.16^***^
Yes	8.20	4.83		12.07	5.38		10.20	5.24		7.32	4.33	
No	11.19	4.72		14.13	2.87		12.54	3.96		8.48	4.67	
Hospital admission			8.24^***^			1.33			2.80^**^			1.57
Yes	9.89	4.85		13.79	3.05		11.79	4.52		8.08	4.45	
No	11.15	4.75		14.13	2.92		12.52	3.99		8.40	4.69	
Usual Source of Care			15.96^***^			2.14^*^			1.94			0.38
Yes	10.33	4.80		13.90	3.07		12.30	4.03		8.32	4.57	
No	12.06	4.57		14.23	2.84		12.60	4.11		8.40	4.90	

*p < 0.05. **p < 0.01. ***p < 0.001

SD, standard deviation

We conducted multiple regression analyses to verify the factors influencing the HL of all subjects and confirmed that HL significantly decreased as age increased (45–64 age group: β=-0.501, *p* < 0.001; 65 years and older group: β=-2.126, *p* < 0.001). Other variables influencing HL included gender, marital status, education level, income level, private health insurance enrollment, walking, SRH, number of chronic diseases, HRQOL, and usual source of care. Specifically, HL was significantly higher for males, individuals with higher income levels, those enrolled in private health insurance, those who practiced walking, individuals with higher SRH status, those with a higher quality of life, and those with a usual source of care. On the other hand, HL was significantly lower for those who were divorced, separated, or widowed, those with lower education levels, and those with a higher number of chronic diseases (Table 3). Detailed statistical results (e.g., β values) are reported in Table 3 for clarity and reference.

**Table 3:** Multiple regression of analysis on health literacy by age group

			Age groups					
	All (*n* = 7,910)		19–44 (*n* = 1,608)		45–64 (*n* = 2,815)		65+ (*n* = 3,487)	
Variable	β	SE	β	SE	β	SE	β	SE
Age (ref = 19–44)								
45–64	-0.501^***^	0.142						
65+	-2.126^***^	0.176						
Gender (ref = female)								
Male	0.380^***^	0.103	0.150	0.167	0.314	0.181	0.456^**^	0.170
Marital Status (ref = not married)								
Married	-0.250	0.169	-0.331^*^	0.154	-0.371	0.368	-0.350	0.744
Divorced/separated/widowed	-0.574^**^	0.201	-0.495	0.446	-0.335	0.400	-0.867	0.746
Education level (ref = college or above)								
Elementary school or below	-3.529^***^	0.156	-10.127^***^	1.423	-3.605^***^	0.286	-3.773^***^	0.283
Middle/high school	-1.065^***^	0.117	-0.456^**^	0.175	-1.086^***^	0.164	-1.586^***^	0.272
Monthly household income (10,000 KRW)	0.002^***^	0.000	0.001^*^	0.0004	0.001^**^	0.0004	0.003^***^	0.001
Healthcare coverage (ref = National Health Insurance)								
Medical Aid	0.195	0.222	-0.830	0.585	-0.119	0.416	0.489	0.313
Having private health insurance (ref = no)								
Yes	1.041^***^	0.113	0.216	0.278	0.861^***^	0.251	1.207^***^	0.150
Economic activity (ref = no)								
Yes	0.045	0.097	0.201	0.161	0.166	0.178	-0.120	0.152
Current smoking (ref = no)								
Yes	-0.116	0.135	-0.384	0.208	-0.089	0.213	-0.019	0.250
High-risk drinking (ref = no)								
Yes	0.002	0.155	0.057	0.210	0.057	0.229	-0.081	0.358
Walking (ref = no)								
Yes	0.250^**^	0.086	0.017	0.142	0.167	0.144	0.404^**^	0.144
Self-rated health (ref = very poor)								
Very good	1.428^**^	0.452	5.892^***^	1.306	1.719	0.996	0.648	0.664
Good	1.921^***^	0.387	5.603^***^	1.275	2.199^*^	0.903	2.006^***^	0.502
Fair	1.726^***^	0.379	5.378^***^	1.272	2.115^*^	0.895	1.676^***^	0.481
Poor	0.774^*^	0.372	5.073^***^	1.285	1.124	0.900	0.665	0.462
Number of chronic diseases (ref = 0)								
1	-0.519^***^	0.128	-0.152	0.197	-0.453^*^	0.185	-1.208^***^	0.289
≥ 2	-0.740^***^	0.139	0.315	0.398	-0.641^**^	0.206	-1.459^***^	0.272
Quality of life (EQ-5D)	2.811^***^	0.524	6.738^***^	1.447	3.126^**^	1.102	2.038^**^	0.698
Disability (ref = no)								
Yes	-0.262	0.171	-0.636	0.584	-0.910*	0.358	-0.035	0.219
Hospital admission (ref = no)								
Yes	0.042	0.125	-0.145	0.252	-0.454*	0.229	0.343	0.182
Number of outpatient visits	-0.001	0.002	0.012^*^	0.006	0.003	0.004	-0.004	0.003
Usual source of care (ref = no)								
Yes	0.364^***^	0.101	-0.223	0.152	0.289	0.163	0.844^***^	0.183
F	197.98^***^		8.26^***^		19.84^***^		46.68^***^	
R^2^	0.386		0.107		0.141		0.237	
R^2^ adjusted	0.384		0.094		0.133		0.232	
AIC	28,887		4,938		10,330		13,305	

*p < 0.05. **p < 0.01. ***p < 0.001

Ref, reference; KRW, Korean won; SE, standard error

The variables consistently associated with HL across all age groups were education level, income level, SRH, and quality of life. We confirmed that the HL level also increased as these variables’ levels increased. On the other hand, marital status was associated with this ability only in the 19–44 age group, while private health insurance enrollment and the number of chronic diseases were associated with HL in the 45–64 and 65 and older age groups. Walking practice and having a usual source of care were associated with HL only in the 65 and older age group (Table 3).

Six variables showed significant associations with HL in the 19–44 age group. HL was lower for individuals with a spouse and those with lower education levels. The better the SRH and the greater the number of outpatient visits, the higher the HL. Eight variables were significantly associated with HL in the 45–64 age group. HL was lower among those with a lower education level. It was higher for those enrolled in private health insurance and for those with SRH of ‘good’ or ‘fair.’ HL was lower among those with a higher number of chronic diseases, those with disabilities, and those who had been hospitalized. Most of the variables associated with HL were in the 65 and older age group, with nine. HL was higher among men, and like other age groups, it was lower for those with lower education levels. It was higher for those enrolled in private health insurance. Individuals who practiced walking demonstrated higher levels of HL. SRH was associated with higher HL for those who rated their health as ‘good’ or ‘fair.’ HL decreased with an increasing number of chronic diseases but was higher for those with a usual source of care (Table 3).

## Discussion

In this study, we measured HL levels across different age groups among 7,910 adults who participated in the health literacy supplement survey of the KHP and explored factors associated with HL in each age group. The findings revealed that HL scores declined as age increased. Younger adults showed ‘adequate’ HL levels; middle-aged adults exhibited ‘problematic’ levels and older adults demonstrated ‘inadequate’ levels when categorized using the HLS-EU-Q16 criteria by Bergman et al. [34]. The multiple regression analysis highlighted that increased age was significantly associated with lower HL levels, aligning with prior research [24, 26, 30, 37–39]. This finding may be explained by age-related declines in sensory functions (e.g., vision and hearing) and cognitive abilities [40], which weakens the ability to comprehend and synthesize health information. Additionally, older adults often face more significant communication barriers than younger age groups [20], such as difficulties understanding healthcare providers’ explanations or written instructions, potentially leading to improper healthcare utilization or mismanagement of their health. Enhancing their HL is crucial to promoting health prevention and disease management among older adults. This will help them better understand their health status and the health information provided by hospitals or pharmacies.

Across all three age groups, education level, income, SRH, and HRQOL were consistently associated with HL. These findings are in line with previous studies reporting that higher educational attainment and income are linked to better HL [22, 24, 27, 41–43], and similarly, better perceived health and quality of life also correlate positively with HL [18, 20, 24, 43, 44]. These common determinants suggest that socioeconomic status and subjective health perception play a significant role in shaping individuals’ ability to understand and utilize health information regardless of age.

This study highlights that HL-related factors differ substantially by age group, underscoring the importance of age-stratified analyses to capture population-specific needs. While education, income, and subjective health indicators were consistently associated with HL across all age groups, several distinct factors emerged in middle-aged and older adults, reflecting changes in health-related circumstances and information-processing demands as people age. Among middle-aged adults, the variables strongly correlated with HL were private health insurance enrollment and the number of chronic diseases. Individuals with private health insurance tend to be more proactive in managing their health and engaging in preventive care. Comparing various insurance plans and selecting the most suitable one may expose individuals to diverse health-related information, enhancing HL. Although this study did not find a significant association between healthcare utilization volume and HL, previous research has suggested that private health insurance enrollment facilitates access to healthcare and promotes system engagement, contributing to improved HL [37, 45]. Conversely, individuals with higher HL may better understand insurance benefits and healthcare options, making them more likely to secure supplemental insurance coverage.

Conflicting research findings have been reported regarding the relationship between HL and the number of chronic diseases. Schillinger et al. and Wolf et al. argued that individuals with more chronic conditions tend to have lower HL [9, 35], whereas Smith et al. found that those with three or more chronic diseases had the highest HL in a sample of adults aged 55–74 [46]. In our study, lower HL was associated with a higher number of chronic diseases, supporting the interpretation that limited HL may hinder disease management and self-care, leading to the accumulation of multiple conditions [47].

In older adults, the significant variables included gender and having a usual source of care. In this study, men’s HL was higher than women’s, aligning with prior research suggesting that older Korean women are more vulnerable in terms of education and social resources, increasing their likelihood of low HL [29]. Other studies have found no gender difference [22, 36, 48] or higher HL in women, possibly due to their greater involvement in family caregiving and frequent use of healthcare services [43, 49]. These conflicting results highlight the influence of demographic and contextual factors in HL disparities. Having a usual source of care was also positively associated with HL among older adults. A usual source of care - defined as a specific healthcare provider or institution regularly visited when medical needs arise [50] - has been shown to enhance access, continuity of care, and adherence to treatment [51, 52]. In the context of aging, where chronic conditions become prevalent and regular care is often needed, having a stable care source may contribute to higher HL by facilitating more precise communication, trust-building, and consistent health engagement.

Taken together, these findings suggest that as age increases, the number and complexity of HL-related factors grow, with a more significant influence from healthcare utilization and health status variables. This may reflect the growing burden of chronic conditions and the increasing need to navigate complex healthcare systems. Age-specific determinants - such as private health insurance in middle-aged adults and gender, walking behavior, and having a usual source of care in older adults - highlight the importance of tailoring HL interventions to each stage of life. Notably, walking was the only health behavior variable associated with HL in the elderly group, suggesting that health-promoting behaviors may also reflect underlying differences in HL [25, 53].

This study has several limitations that should be acknowledged. First, it employed a cross-sectional design using the 2021 KHP health literacy supplement survey, which limits causal inference due to the absence of temporal sequencing and restricts the ability to explore potential causal mechanisms. Second, due to data limitations, psychosocial factors such as self-efficacy and social support, which are known to influence HL, were not included in the analysis. Third, the HLS-EU-Q16 used in this study is a subjective, self-reported tool that may reflect perceived rather than actual HL. In addition, the instrument is more focused on healthcare and disease prevention than health promotion, which may result in an incomplete assessment of HL, particularly in capturing preventive and proactive aspects. Finally, the data were collected during the COVID-19 pandemic, and restrictions on face-to-face interactions may have influenced healthcare utilization and HL behaviors. Further research could employ a longitudinal design to capture the dynamic process of HL changes over time and establish causal relationships. Moreover, incorporating psychosocial factors and using measurement tools that cover multiple domains, including health promotion, disease prevention, and healthcare, would significantly enhance the assessment of the multifaceted influences on HL. Despite these limitations, this study has important strengths, including the use of a nationally representative sample of nearly 8,000 individuals and a validated HL measurement tool (HLS-EU-Q) designed for the general population [5], which together provide a valuable basis for identifying age-specific HL determinants and informing targeted interventions.

## Conclusion

We found that HL significantly decreases with increasing age among the general adult population in Korea. Notably, older adults exhibited the lowest levels of HL. In addition, factors related to HL differed by age group. These findings highlight the need for targeted interventions that address the specific needs of different age groups. Interventions to improve HL are significant for aging populations facing distinct health needs, such as managing chronic diseases and maintaining functional independence. For older adults, it is necessary to implement interventions that consider other factors that significantly influence this ability, such as educational level, health behaviors like regular physical activity, and access to regular healthcare services. Specifically, strategies that promote regular physical activity and ensure consistent access to healthcare providers may prove most effective. HL interventions addressing the specific needs of each age group could help reduce health disparities and improve overall health outcomes.

## Electronic supplementary material

Below is the link to the electronic supplementary material.

Supplementary Material 1

Supplementary Material 2

## Data Availability

The raw data supporting this study’s findings are publicly available from the Korea Health Panel Survey with permission. However, data availability for processed data is limited because, according to the bylaws of the Korea Health Panel Survey, the free sharing of data between individuals is prohibited. The processed data is available upon reasonable request and with the permission of the National Health Insurance Services and the Korea Institute for Health and Social Affairs.

## Abbreviations

HL: Health literacy
KHP: Korea Health Panel
NHI: National Health Insurance
SRH: Self-rated health
EQ-5D: EuroQoL 5 Dimension
HLS-EU-Q: The European Health Literacy Survey Questionnaire
HRQOL: Health-related quality of life

## References

[1] Ratzan SC, Parker RM. Introduction. In: Selden CR, Zorn M, Ratzan SC, Parker RM, editors. National library of medicine current bibliographies in medicine: health literacy. Bethesda, MD: National Institutes of Health; 2000. (NLM Pub. No. CBM 2000–1).

[2] Baker DW, Williams MV, Parker RM, Gazmararian JA, Nurss J. Development of a brief test to measure functional health literacy. Patient Educ Couns. 1999;38(1):33–42. 10.1016/s0738-3991(98)00116-5. 14528569 10.1016/s0738-3991(98)00116-5

[3] Davis TC, Long SW, Jackson RH, Mayeaux EJ, George RB, Murphy PW, Crouch MA. Rapid estimate of adult literacy in medicine: a shortened screening instrument. Fam Med. 1993;25(6):391–5. PMID: 8349060. 8349060

[4] Sun X, Shi Y, Zeng Q, Wang Y, Du W, Wei N, Xie R, Chang C. Determinants of health literacy and health behavior regarding infectious respiratory diseases: a pathway model. BMC Public Health. 2013;13:261. 10.1186/1471-2458-13-261. 23521806 10.1186/1471-2458-13-261 PMC3621712

[5] Gustafsdottir SS, Sigurdardottir AK, Arnadottir SA, Heimisson GT, Mårtensson L. Translation and cross-cultural adaptation of the European health literacy survey questionnaire, HLS-EU-Q16: the Icelandic version. BMC Public Health. 2020;20(1):61. 10.1186/s12889-020-8162-6. 31937293 10.1186/s12889-020-8162-6 PMC6961374

[6] Berkman ND, Sheridan SL, Donahue KE, Halpern DJ, Crotty K. Low health literacy and health outcomes: an updated systematic review. Ann Intern Med. 2011;155(2):97–107. 10.7326/0003-4819-155-2-201107190-00005. 21768583 10.7326/0003-4819-155-2-201107190-00005

[7] Nutbeam D. The evolving concept of health literacy. Soc Sci Med. 2008;67(12):2072–8. 10.1016/j.socscimed.2008.09.050. 18952344 10.1016/j.socscimed.2008.09.050

[8] Ishikawa H, Yano E. Patient health literacy and participation in the health-care process. Health Expect. 2008;11(2):113–22. 10.1111/j.1369-7625.2008.00497.x. 18494956 10.1111/j.1369-7625.2008.00497.x PMC5060442

[9] Schillinger D, Grumbach K, Piette J, Wang F, Osmond D, Daher C, Palacios J, Sullivan GD, Bindman AB. Association of health literacy with diabetes outcomes. JAMA. 2002;288(4):475–82. 10.1001/jama.288.4.475. 12132978 10.1001/jama.288.4.475

[10] Jayasinghe UW, Harris MF, Parker SM, Litt J, van Driel M, Mazza D, Del Mar C, Lloyd J, Smith J, Zwar N, Taylor R. Preventive evidence into practice (PEP) partnership group. The impact of health literacy and life style risk factors on health-related quality of life of Australian patients. Health Qual Life Outcomes. 2016;14:68. 10.1186/s12955-016-0471-1. 27142865 10.1186/s12955-016-0471-1 PMC4855442

[11] Sansom-Daly UM, Lin M, Robertson EG, et al. Health literacy in adolescents and young adults: an updated review. J Adolesc Young Adult Oncol. 2016;5(2):106–18. 10.1089/jayao.2015.0059. 26859721 10.1089/jayao.2015.0059

[12] Baker DW, Wolf MS, Feinglass J, et al. Health literacy and mortality among elderly persons. Arch Intern Med. 2007;167(14):1503–9. 10.1001/archinte.167.14.1503. 17646604 10.1001/archinte.167.14.1503

[13] Davis TC, Wolf MS, Bass PF 3rd, Middlebrooks M, Kennen E, Baker DW, Bennett CL, Durazo-Arvizu R, Bocchini A, Savory S, Parker RM. Low literacy impairs comprehension of prescription drug warning labels. J Gen Intern Med. 2006;21(8):847–51. 10.1111/j.1525-1497.2006.00529.x. 16881945 10.1111/j.1525-1497.2006.00529.x PMC1831578

[14] Scott TL, Gazmararian JA, Williams MV, Baker DW. Health literacy and preventive health care use among medicare enrollees in a managed care organization. Med Care. 2002;40(5):395–404. 10.1097/00005650-200205000-00005. 11961474 10.1097/00005650-200205000-00005

[15] Institute of Medicine (US) Committee on Health Literacy; Nielsen-Bohlman L, Panzer AM, Kindig DA, editors. Health Literacy: A Prescription to End Confusion. Washington (DC): National Academies Press (US). 2004. 3, The Extent and Associations of Limited Health Literacy. https://www.ncbi.nlm.nih.gov/books/NBK216036/ Accessed 20 Aug 2024. 25009856

[16] Manganello JA, Sojka CJ. An exploratory study of health literacy and African American adolescents. Compr Child Adolesc Nurs. 2016;39(3):221–39. 10.1080/24694193.2016.1196264.

[17] Cha E, Kim KH, Lerner HM, Dawkins CR, Bello MK, Umpierrez G, Dunbar SB. Health literacy, self-efficacy, food label use, and diet in young adults. Am J Health Behav. 2014;38(3):331–9. 10.5993/AJHB.38.3.2. 24636029 10.5993/AJHB.38.3.2 PMC4039409

[18] Lee SY, Tsai TI, Tsai YW, Kuo KN. Health literacy, health status, and healthcare utilization of Taiwanese adults: results from a National survey. BMC Public Health. 2010;10:614. 10.1186/1471-2458-10-614. 20950479 10.1186/1471-2458-10-614 PMC2967535

[19] Cutilli CC. Health literacy in geriatric patients: an integrative review of the literature. Orthop Nurs. 2007;26(1):43–8. 10.1097/00006416-200701000-00014. 17273109 10.1097/00006416-200701000-00014

[20] Gray NJ, Klein JD, Noyce PR, Sesselberg TS, Cantrill JA. Health information-seeking behaviour in adolescence: the place of the internet. Soc Sci Med. 2005;60(7):1467–78. 10.1016/j.socscimed.2004.08.010. 15652680 10.1016/j.socscimed.2004.08.010

[21] Cordasco KM, Asch SM, Franco I, Mangione CM. Health literacy and english Language comprehension among elderly inpatients at an urban safety-net hospital. J Health Hum Serv Adm. 2009;32(1):30–50. PMID: 19558032. 19558032

[22] Wister AV, Malloy-Weir LJ, Rootman I, Desjardins R. Lifelong educational practices and resources in enabling health literacy among older adults. J Aging Health. 2010;22(6):827–54. 10.1177/0898264310373502. 20595098 10.1177/0898264310373502

[23] Song I. Relationship between health literacy and health-related quality of life in Korean adults with chronic diseases. PLoS ONE. 2024;19(4):e0301894. 10.1371/journal.pone.0301894. 38635779 10.1371/journal.pone.0301894 PMC11025905

[24] Wolf MS, Gazmararian JA, Baker DW. Health literacy and functional health status among older adults. Arch Intern Med. 2005;165(17):1946–52. 10.1001/archinte.165.17.1946. 16186463 10.1001/archinte.165.17.1946

[25] Al Sayah F, Johnson ST, Vallance J. Health literacy, pedometer, and Self-Reported walking among older adults. Am J Public Health. 2016;106(2):327–33. 10.2105/AJPH.2015.302901. 26691129 10.2105/AJPH.2015.302901 PMC4815562

[26] Eronen J, Paakkari L, Portegijs E, Saajanaho M, Rantanen T. Assessment of health literacy among older Finns. Aging Clin Exp Res. 2019;31(4):549–56. 10.1007/s40520-018-1104-9. 30578457 10.1007/s40520-018-1104-9 PMC6439255

[27] Peterson PN, Shetterly SM, Clarke CL, Bekelman DB, Chan PS, Allen LA, Matlock DD, Magid DJ, Masoudi FA. Health literacy and outcomes among patients with heart failure. JAMA. 2011;305(16):1695–701. 10.1001/jama.2011.512. 21521851 10.1001/jama.2011.512 PMC4540335

[28] Zou H, Chen Y, Fang W, Zhang Y, Fan X. The mediation effect of health literacy between subjective social status and depressive symptoms in patients with heart failure. J Psychosom Res. 2016;91:33–9. 10.1016/j.jpsychores.2016.10.006. 27894460 10.1016/j.jpsychores.2016.10.006

[29] Kwon MS, Noh GY, Jang JH. A study on relationships between health literacy, Disease-related knowledge and compliance to medical recommendations in patients with hypertension. J Korean Public Health Nurs. 2013;27(1):190–202. 10.5932/jkphn.2013.27.1.190.

[30] Lorini C, Lastrucci V, Mantwill S, Vettori V, Bonaccorsi G, Florence Health Literacy Research Group. Measuring health literacy in Italy: a validation study of the HLS-EU-Q16 and of the HLS-EU-Q6 in Italian Language, conducted in Florence and its surroundings. Ann Ist Super Sanita. 2019;55(1):10–8. 10.4415/ANN_19_01_04. 30968831 10.4415/ANN_19_01_04

[31] Kim DS, Sung SH, Shin S, Park M. The effect of cancer on traditional, complementary and alternative medicine utilization in Korea: a fixed effect analysis using Korea health panel data. BMC Complement Med Ther. 2022;22:137. 10.1186/s12906-022-03614-0. 35585580 10.1186/s12906-022-03614-0 PMC9118572

[32] Choi SK. A Study for Improving Health Literacy (KIHASA Report No. 20–24). Korea Institute for Health and Social Affairs. 2020. https://www.kihasa.re.kr/en/publish/paper/research/view?seq=35642 Accessed 1 Sep 2024.

[33] Pelikan JM, Ganahl K. Measuring health literacy in general populations: primary findings from the HLS-EU consortium’s health literacy assessment effort. Stud Health Technol Inf. 2017;240:34–59. PMID: 28972508. 28972508

[34] Bergman L, Nilsson U, Dahlberg K, Jaensson M, Wångdahl J. Validity and reliability of the Arabic version of the HLS-EU-Q16 and HLS-EU-Q6 questionnaires. BMC Public Health. 2023;23(1):304. 10.1186/s12889-023-15226-5. 36765302 10.1186/s12889-023-15226-5 PMC9912492

[35] Wolf MS, Feinglass J, Thompson J, Baker DW. In search of ‘low health literacy’: threshold vs. gradient effect of literacy on health status and mortality. Soc Sci Med. 2010;70(9):1335–41. 10.1016/j.socscimed.2009.12.013. 20167411 10.1016/j.socscimed.2009.12.013

[36] Kim SH, Lee E. The influence of functional literacy on perceived health status in Korean older adults. Taehan Kanho Hakhoe Chi. 2008;38(2):195–203. 10.4040/jkan.2008.38.2.195. 18458516 10.4040/jkan.2008.38.2.195

[37] Lee MS. Health literacy and health behaviors among older adults with Cardio-cerebrovascular disease residing in rural areas. Korean J Adult Nurs. 2017;29(3):256–65. 10.7475/kjan.2017.29.3.256.

[38] Wei MH. The associations between health literacy, reasons for seeking health information, and information sources utilized by Taiwanese adults. Health Educ J. 2014;73(4):423–34. 10.1177/0017896912471523.

[39] Peng Y, Wang X, Li P, Liu K. Mental health literacy in Changsha, China. Shanghai Arch Psychiatr. 2011;23(6):353–9. 10.3969/j.issn.1002-0829.2011.06.005.

[40] Safeer RS, Keenan J. Health literacy: the gap between physicians and patients. Am Fam Physician. 2005;72(3):463–8. PMID: 16100861. 16100861

[41] Ganzer CA, Insel KC, Ritter LS. Associations between working memory, health literacy, and recall of the signs of stroke among older adults. J Neurosci Nurs. 2012;44(5):236–43. 10.1097/JNN.0b013e3182666231. 22955236 10.1097/JNN.0b013e3182666231

[42] von Wagner C, Knight K, Steptoe A, Wardle J. Functional health literacy and health-promoting behaviour in a National sample of British adults. J Epidemiol Community Health. 2007;61(12):1086–90. 10.1136/jech.2006.053967. 18000132 10.1136/jech.2006.053967 PMC2465677

[43] Sudore RL, Mehta KM, Simonsick EM, Harris TB, Newman AB, Satterfield S, Rosano C, Rooks RN, Rubin SM, Ayonayon HN, Yaffe K. Limited literacy in older people and disparities in health and healthcare access. J Am Geriatr Soc. 2006;54(5):770–6. 10.1111/j.1532-5415.2006.00691.x. 16696742 10.1111/j.1532-5415.2006.00691.x

[44] Zheng M, Jin H, Shi N, Duan C, Wang D, Yu X, Li X. The relationship between health literacy and quality of life: a systematic review and meta-analysis. Health Qual Life Outcomes. 2018;16(1):201. 10.1186/s12955-018-1031-7. 30326903 10.1186/s12955-018-1031-7 PMC6192335

[45] Vera-Hernández AM. Duplicate coverage and demand for health care. The case of Catalonia. Health Econ. 1999;8(7):579–98. 10.1002/(sici)1099-1050(199911)8:7%3C579::aid-hec478%3E3.0.co;2-p. 10.1002/(sici)1099-1050(199911)8:7<579::aid-hec478>3.0.co;2-p 10544325

[46] Smith SG, Curtis LM, Wardle J, von Wagner C, Wolf MS. Skill set or Mind set? Associations between health literacy, patient activation and health. PLoS ONE. 2013;8(9):e74373. 10.1371/journal.pone.0074373. 24023942 10.1371/journal.pone.0074373 PMC3762784

[47] Greene J, Hibbard JH, Tusler M. How much do health literacy and patient activation contribute to older adults’ ability to manage their health? AARP Public Policy Institute. 2005. https://digirepo.nlm.nih.gov/master/borndig/101259851/2005_05_literacy.pdf Accessed 3 Sep 2024.

[48] Patel PJ, Joel S, Rovena G, Pedireddy S, Saad S, Rachmale R, Shukla M, Deol BB, Cardozo L. Testing the utility of the newest vital sign (NVS) health literacy assessment tool in older African-American patients. Patient Educ Couns. 2011;85(3):505–7. 10.1016/j.pec.2011.03.014. 21514089 10.1016/j.pec.2011.03.014

[49] Lee JE, Lee SY, Noh HK, Lee E. The influence of functional health literacy on health promotion behavior. J Korean Data Inf Sci Soc. 2015;26(6):1427–38. 10.7465/jkdi.2015.26.6.1427.

[50] Kim D, Sung NJ. Types of usual source of care and Patient-Centered communications. Korean J Fam Med. 2022;43(6):353–60. 10.4082/kjfm.21.0183. 36444119 10.4082/kjfm.21.0183 PMC9708853

[51] Kim KM, Kim CY. The association between types of usual source of care and unmet health care needs. Health Soc Sci. 2020;53(1):105–28. 10.37243/kahms.2020.53.105.

[52] Jung Y, Byeon J. The association between having a usual source of care and adherence to medicines in patients with chronic diseases. Korean J Clin Pharm. 2016;26(2):128–36. https://pesquisa.bvsalud.org/portal/resource/pt/wpr-121732.

[53] Martin LT, Haas A, Schonlau M, Derose KP, Rosenfeld L, Rudd R, Buka SL. Which literacy skills are associated with smoking? J Epidemiol Community Health. 2012;66(2):189–92. 10.1136/jech.2011.136341. 22003080 10.1136/jech.2011.136341 PMC3246123

