# Supplementary material for: Patterns of health literacy and influencing factors differ by age: a cross-sectional study
Source: BMC Public Health. 2025 Apr 26;25:1556. doi: 10.1186/s12889-025-22838-6 (PMC12032677; doi:10.1186/s12889-025-22838-6)
Supplement: Supplementary file 1 — Supplementary Material 1 [file 12889_2025_22838_MOESM1_ESM.docx]

**Domains and Items of HLS-EU-Q16**

| **Domain** | **Item** |
| --- | --- |
| **Healthcare** | 1. find information on treatments of illnesses that concern you? |
|  | 2. find out where to get professional help when you are ill? |
|  | 3. understand what your doctor says to you? |
|  | 4. understand your doctor’s or pharmacist’s instruction on how to take a prescribed medicine? |
|  | 5. judge when you may need to get a second opinion from another doctor? |
|  | 6. use information the doctor gives you to make decisions about your illness? |
|  | 7. follow instructions from your doctor or pharmacist? |
| **Disease Prevention** | 8. find information on how to manage mental health problems like stress of depression? |
|  | 9. understand health warnings about behavior such as smoking, low physical activity and drinking too much? |
|  | 10. understand why you need health screenings? |
|  | 11. judge if the information on health risks in the media is reliable? |
|  | 12. decide how you can protect yourself from illness based on information in the media? |
| **Health Promotion** | 13. find out about activities that are good for your mental well-being? |
|  | 14. understand advice on health from family members or friends? |
|  | 15. understand information in the media on how to get healthier? |
|  | 16. judge which everyday behavior is related to your health? |
