# Supplementary figures and images for: Patterns of health literacy and influencing factors differ by age: a cross-sectional study
Source: BMC Public Health. 2025 Apr 26;25:1556. doi: 10.1186/s12889-025-22838-6 (PMC12032677; doi:10.1186/s12889-025-22838-6)

**2020-2021 Annual Data Structure**

**
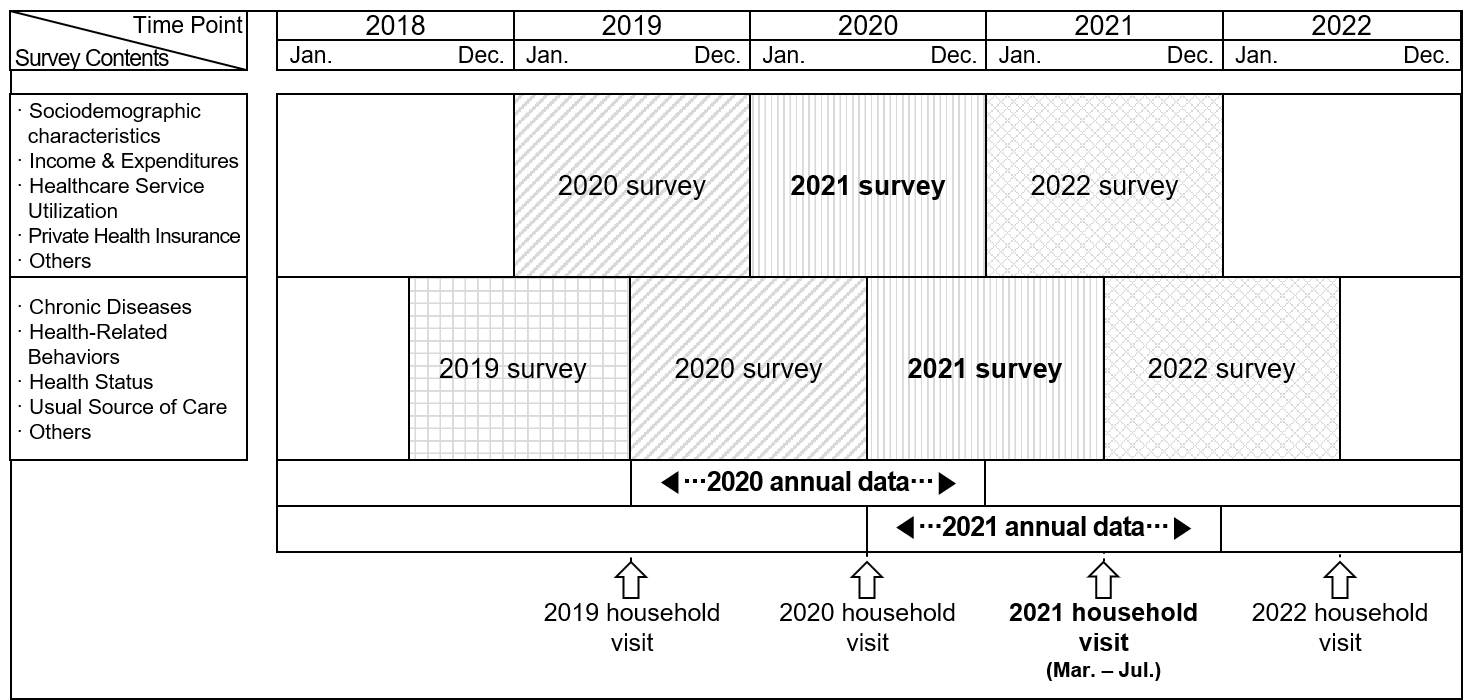
**

Supplement: Supplementary file 2 — Supplementary Material 2 [file 12889_2025_22838_MOESM2_ESM.docx]
